# Promoting healthy eating and physical activity among school children: findings from Health-E-PALS, the first pilot intervention from Lebanon

**DOI:** 10.1186/1471-2458-14-940

**Published:** 2014-09-10

**Authors:** Carla Habib-Mourad, Lilian A Ghandour, Helen J Moore, Maya Nabhani-Zeidan, Kassim Adetayo, Nahla Hwalla, Carolyn Summerbell

**Affiliations:** Department of Nutrition and Food Sciences, Faculty of Agriculture and Food Sciences, American University of Riad El-Solh, PO Box 11–0236, Beirut, 1107-2020 Lebanon; School of Medicine, Pharmacy and Health, Durham University, Stockton-on-Tees, UK; Department of Epidemiology and Population Health, American University of Beirut, Beirut, Lebanon

**Keywords:** Childhood obesity, School-based interventions, Eastern Mediterranean region, Pilot trial

## Abstract

**Background:**

In Lebanon, childhood obesity doubled during the past decade. Preventive measures should start early in life and Schools are considered an important environment to promote energy balance health behaviours. School-based programmes promoting healthy lifestyles are lacking. The purpose of this study was to evaluate the feasibility and effectiveness of a multicomponent school-based intervention to promote healthy eating and physical activity (and prevent obesity) with school children aged 9–11 years in Lebanon.

**Methods:**

The intervention was developed based on the constructs of the Social Cognitive Theory and adapted to the culture of Lebanese and Arab populations. It consisted of three components: class curriculum, family involvement and food service. Eight schools were purposively selected from two communities of different socioeconomic status (SES) in Beirut and, within each school type, were matched on SES, religious sect profile, and then randomly assigned to either the intervention or control group. Anthropometric measurements and questionnaires on determinants of behavioural change, eating and physical activity habits were completed by the students in both groups at baseline and post intervention. Focus group interviews were conducted in intervention schools at the end of the study. Challenges encountered during the programme implementation were also identified, since Lebanon is considered a country with political unrest and no similar research projects were conducted in the area.

**Results:**

Students in the intervention group reported purchasing and consuming less chips and sweetened drinks post-intervention compared with controls (86% & 88% less respectively *p* < 0.001). Knowledge and self-efficacy scores increased for the intervention (+2.8 & +1.7 points respectively *p* < 0.001) but not for the control group. There was no difference in physical activity and screen time habits and no changes in BMI between groups at post intervention. Interview data from focus groups showed that the programme was generally well accepted. Limitations for better outcomes include the length of the programme and the school environment.

**Conclusion:**

“Health-E-PALS” intervention is a promising innovative, theory-based, culturally sensitive intervention to promote healthy eating habits and physical activity in Lebanese school children with a potential to be scaled up, replicated and sustained.

**Electronic supplementary material:**

The online version of this article (doi:10.1186/1471-2458-14-940) contains supplementary material, which is available to authorized users.

## Background

Overweight and obesity are serious problems posing one of the most difficult public health challenges of the 21st century in many countries. The Middle East and the Arabian Peninsula regions, which include Lebanon, are not exempt from the obesity epidemic, which has reached worrying levels both among children and adults [1].

Lebanon is a small middle-income country characterised by a high urbanisation rate (81%), a high literacy rate (85%) and a life expectancy close to 72 years [2]. A monitoring study reporting on overweight and obesity trends in Lebanon in 1997 and 2009 showed a rapid increase in body mass index (BMI) across sex and age groups, particularly 6–19 year olds [3]. This study also showed that childhood obesity which continues through adolescence occurs before age 11 [4]. Children aged 13 to 15 years reported poor eating habits including skipping breakfast, a low intake of fruits and vegetables, and a high consumption of high energy foods and beverages, compared with recommended [5]. Coupled with the low physical activity levels reported by these children, these risk factors help explain the increased prevalence of overweight and obesity in Lebanon [3, 6, 7].

In response, policies and strategies have been recommended to promote weight control and physical activity across Lebanon [8, 9]. But school-based interventions promoting healthy eating and physical activity are still lacking, and the Lebanese integrated health curriculum incorporates very little nutrition education. Several reviews have assessed the effectiveness of school-based interventions in preventing and reducing childhood obesity [10–12]. However, most of the interventions have been conducted in North America, Australia and Europe; very little research in this area has been carried out in the Middle-Eastern region. Although certain key concepts and elements of the successful interventions identified in these reviews can be translated for the Lebanese population, such as the importance of using a whole school-based approach [10] which targets behaviour change at the individual level and also changing the school food environment, new or adapted interventions require pilot testing because of the distinct Lebanese cultural context.

In this realm, this study was undertaken to fulfil two objectives: (1) To develop a theory and evidence-based multi-component school intervention that is culturally appropriate, which takes a whole school-based approach, and is participatory-based (involving the school environment and parents), that aims to promote healthy eating and physical activity among children aged 9–11 years; and (2) by conducting a pilot study, to evaluate the feasibility and effectiveness of this multicomponent school-based intervention to promote healthy eating and physical activity (and prevent obesity) in school children aged 9–11 years in Lebanon.

This paper reports on the process and outcomes of the pilot study. A paper reporting on the development of the intervention is published elsewhere [13].

## Methods

### Study design

This pilot/feasibility study used a sequential explanatory mixed method study design, involving both quantitative and qualitative research methods.

### Study site and population

The study was conducted in Beirut, the largest and capital city of Lebanon, comprised of a mélange of ethnic and religious sects. Following ethical approval by the Institutional Review Board (IRB) at the American University of Beirut, we contacted the Ministry of Education, whose support helped facilitate the process of recruitment. Both private and public primary schools were sampled (primary sampling units) to include students of various socioeconomic levels, since middle-high income families in Lebanon tend to enrol their children in private schools given the high annual tuition fees, and lower income families tend to send their children to public schools for a nominal fee. The schools were purposively selected to include socioeconomically and religiously diverse catchment areas.

**Figure 1:**
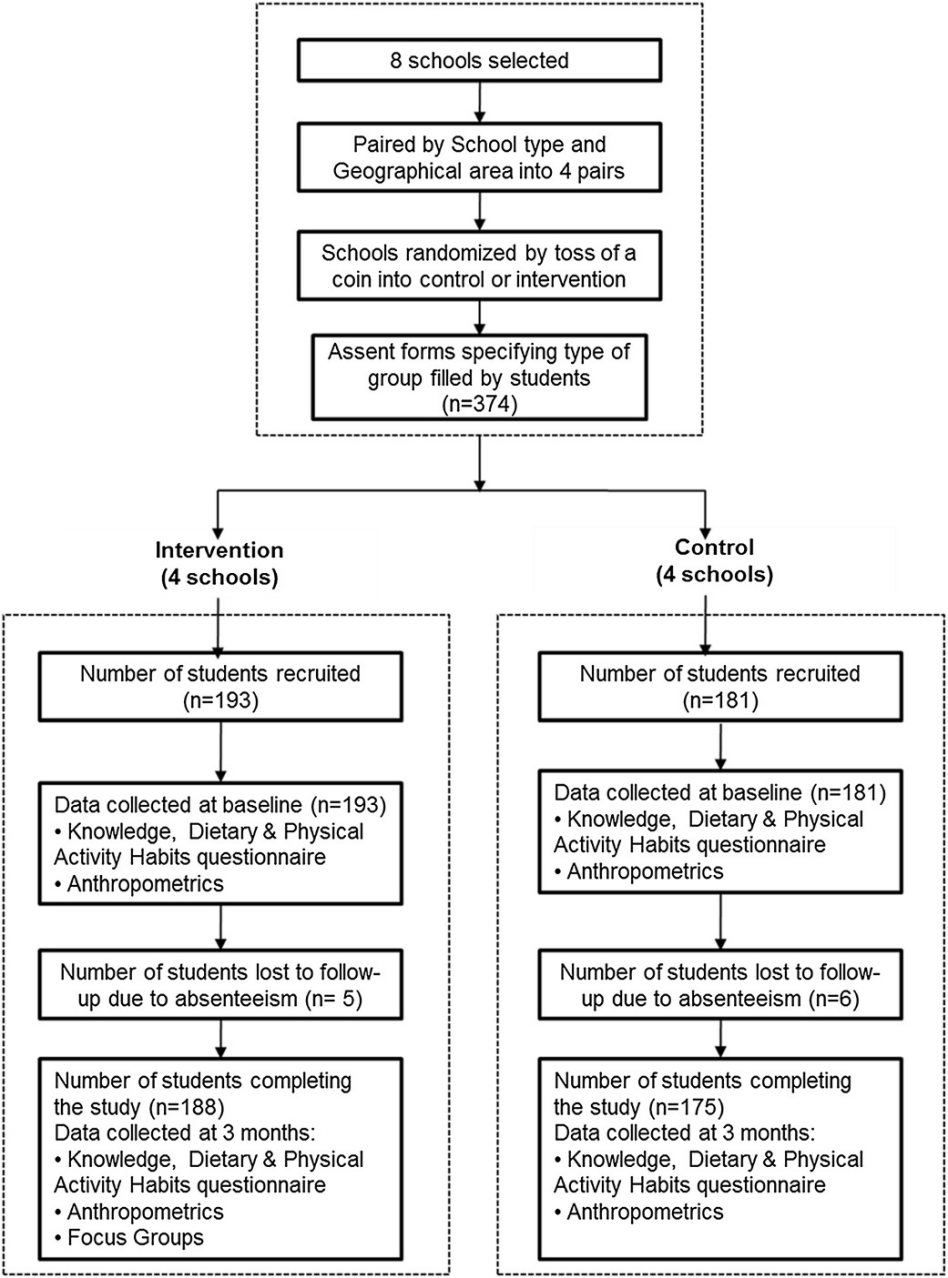
**Students flow diagram.**

For this pilot study, four private and four public schools were purposively selected. The four private schools were then assigned to matched pairs, according to their socioeconomic status based on neighbourhood, and religious sect. The same principles of allocation were applied to the four public schools. Then, within each matched pair, one school was randomly assigned (by the toss of a coin) to receive the intervention, and the other school served as the control. Ultimately, four schools received the intervention (2 private and 2 public) and four others were control schools. Despite the limited number of schools (n = 8), the method of randomization distributed potential confounders equally between intervention and control groups, and students’ baseline characteristics in their respective school pairs were comparable (Table 1).All students in Grades 4 and 5 (aged 9–11 years) were invited to take part in the pilot study. Consent forms were sent to the students’ parents/guardians to obtain their approval, students also signed assent forms. All students accepted to participate resulting in a total sample of 387 students agreeing to partake in the study; after school randomization, a total of 193 students were assigned to receive the intervention and 181 served as control (i.e. received the usual curriculum during the pilot study period). Figure 1 is a flow diagram illustrating the progress of student recruitment and data collection in both groups from baseline to study completion.

**Table 1:** **General characteristics of students and schools included in the programme**

	School pair 1		School pair 2		School pair 3		School pair 4		Total Schools	
	Intervention	Control	Intervention	Control	Intervention	Control	Intervention	Control	Intervention	Control
**No. of students**	21	23	79	66	51	56	41	37	193	181
**School Type**	Private	Private	Private	Private	Public	Public	Public	Public	4	4
**Average Students /class**	21	23	26	22	25	28	20	19	23	23
**Gender**										
Male% (n)	38 (8)	52 (12)	55(43)	52 (39)	59 (30)	55 (31)	71 (29)	46 (17)	57(111)	53(93)
Female% (n)	62(13)	48 (11)	45 (36)	48 (27)	41 (21)	45 (25)	29 (12)	54 (20)	43(82)	47(88)
	**Mean SD**	**Mean SD**	**Mean SD**	**Mean SD**	**Mean SD**	**Mean SD**	**Mean SD**	**Mean SD**	**Mean SD**	**Mean SD**
**Age (yrs.)**	9.6 0.6	9.3 0.5	10.4 0.5	10.2 3.5	10.6 1.2	10.4 1.2	10.4 1.2	9.9 1.1	10.3 0.9	10.1 1
**BMI**	20.2 3.6	9.3 0.5	20.8 4	19.9 3.5	18.9 3.8	17.3 3.2	18.4 3.8	18.5 3.3	19.7 4	18.8 3.5
**Waist circumference**	69.9 9.2	66.8 7.8	75.1 11.5	72.6 9.7	68.8 8.7	65 9.1	65.7 8.5	68 8.7	70.9 1.1	69.8 1

### Intervention design

The development and design of the intervention are described briefly in this section, more details are found elsewhere [13]. The intervention used a ‘whole school approach’, which targeted both individual (children’s) behavior change and also the school environment.

Students in the intervention schools received the programme components over three consecutive months; students in the control schools received their usual curriculum during the intervention period. The school-based multicomponent intervention was initially called ‘Kanz al Soha’ in Arabic which translates to “The treasure of Health”. Subsequently, ‘Health-E-PALS’ was deduced as the acronym for: ‘Intervention to promote Healthy Eating and Physical Activity in Lebanese School children’. The intervention specifically targeted obesity-related behaviours in 9–11 year olds including: increasing consumption of fruits and vegetables, favouring healthy over high energy dense snacks and drinks, increasing the habit of having breakfast daily, increasing moderate-to-vigorous physical activity (MVPA), and decreasing overall sedentary behaviour.

The ‘Health-E-PALS’ intervention was based on the constructs of the Social Cognitive Theory [14], which uses a multilevel approach involving individual behaviour change and environment modifications to support individual changes. Role modelling of significant others and availability of healthy choices at home and school were the main environmental factors addressed by the programme.

Specifically, the intervention was comprised of 3 coordinated components. First, 12 culturally appropriate classroom sessions using fun and interactive activities were delivered once a week for 3 consecutive months. The activities were incorporated into the school curriculum and delivered mainly by the first author of this paper (Carla Habib-Mourad), a specialist in community nutrition, with the support of one research assistant who is also a nutritionist. Second, a family programme consisting of meetings, health fairs as well as information packets was sent home along with some food samples and recipes. Lastly, a food service intervention targeted the school shops and the lunch boxes sent by the family.

### Statistical methods

#### Quantitative data collection and analysis

For students in both intervention and control schools, a baseline assessment (pre-test) was conducted a week prior to the start of the intervention, followed by another assessment one week after the completion of the intervention (post-test). Both assessments took place in the classroom. Anthropometric measurements including height, weight, and waist circumference were carried out at both time points using standardized techniques and calibrated equipment (Seca balance and Stadiometer model 11770 Germany, and plastic measuring tape).

The questionnaire used for both the pre- and post-tests was designed to provide an indication of dietary, physical activity, and sedentary behaviour habits. The questionnaire was not designed to measure dietary intake, MVPA, or sedentary behaviours. The questionnaire included three sections (1) dietary habits (13 questions), questions included regularity of intake of meals, types of snacks eaten or bought from the school shop, frequency of eating out, and eating in front of TV; (2) physical activity/sedentary habits (10 questions) questions included regularity of sessions of physical education at school, playing outdoors at school and at home, after school structured activity per week, watching TV, playing computer games during week days and week-ends; (3) nutrition knowledge (14 questions) and self-efficacy (9 questions). The questions and questionnaire (Additional file 1) were adapted from a previously developed questionnaire used in Lebanese children [8].

The questions on dietary and physical activity behaviours were individually analysed. For knowledge questions, each response was recoded as either 1 (correct answer) or 0 (for an incorrect answer, including the response ‘don’t know’), and summed to generate a single score (range: 0–14) reflecting overall level of knowledge; the higher the score the better the knowledge. The 9 self-efficacy items were also summed into a single score (range 0–18); each question was measured on a 3-point Likert scale (0 = not sure, 1 = little sure, 2 = very sure); the higher the score, the better the self-efficacy. The internal consistency (and item-total correlations) of each set of knowledge and self-efficacy items was checked prior to creation of the overall scores; in both cases, Cronbach’s alpha was 0.66 at pre assessment and 0.7 at post assessment.

Exploratory data analysis was initially conducted and descriptive statistics were calculated using Pearson‘s Chi-square tests and reported for all major study variables. Observed and relative frequencies are reported for categorical variables, and measures of tendency and deviation were calculated for continuous data. Regression models were then estimated, particularly Generalized Estimated Equations (GEE), in order to generate population-averaged estimates. Given that students were clustered within each school, robust standard errors were calculated to account for non-independence of student observations within each school (cluster = school). Paired t-tests were used to assess the differences in means between pre and post surveys of each group alone. Independent t-tests assessed mean change differences between intervention and control groups; the normality assumption for all continuous variables was checked and satisfied prior to undertaking the test. After recoding categorical variables to binary format (recoding methods are detailed in Additional file 2), GEE with a logit link for binary data was carried out to generate the odds ratios comparing diet and physical activity habits between children in intervention schools compared with control schools, and thus the impact of the intervention on these behaviours.

### Qualitative data collection and analysis

Focus group discussions (FGDs) were conducted with a sub-sample of the students, their parents and teachers in intervention schools only, after having completed the intervention sessions and received the post-test. Parents, students and teachers’ FGDs were held separately, and lasted approximately 45 minutes each. A total of 13 focus groups and two in-depth interviews with a total number of 82 participants were completed. The aim of the FGDs and interviews was to estimate the children’s, parents’ and teachers’ overall perception of the programme, what they liked and did not like, to understand in depth what the students felt they have gained, and to further explore and help explain the quantitative outcomes resulting from the intervention.

All FGDs and interviews were carried out in colloquial Arabic, audio taped following the participants’ permission, transcribed verbatim in Arabic and analysed using thematic content analysis, a method described by Burnard [15]. Thematic content analysis is adapted from grounded theory, and was carried out by the first author (Carla Habib-Mourad) using the systematic approach of immersion in the data, coding, and data reduction into themes. The researcher analysed the data and identified categories, which were then compared and contrasted to arrive at the major themes.

### Process evaluation

A process evaluation was conducted to explore the implementation, receipt, and setting of the intervention and help in the interpretation of the outcome results. It aimed to examine the views of participants, parents and teachers who were involved with the intervention; study how the intervention was implemented and whether it was delivered as intended; distinguish between components of the intervention; investigate contextual factors that affected the intervention; and monitor the reach of the intervention;

Data collection was through direct researchers’ observation and field notes, (from teachers and parents comments that were collected during family events and school visits), and from focus group interviews.

## Results

### Changes in dietary habits

Table 2 presents the baseline and post measures of students dietary, physical activity and sedentary behaviours in both intervention and control schools. Students belonging to both groups (intervention or control) were very similar (p > 0.05) in their baseline behaviours and habits, while marked differences were observed at post-test (p *<* 0.05).

**Table 2:** **Baseline and post measures for dietary, physical activity, sedentary habits, knowledge and self-efficacy in intervention and control schools**

	Baseline measures		Post- measures	
	Intervention	Control	Intervention	Control
	(n = 193)	(n = 181)	(n = 193)	(n = 181)
	% (n)			
**Dietary habits**				
Breakfast intake	71.4(137)	63.0(114)	76.5 (143)	48.0 (84)
Number of snacks per day (3 or more)	44.1(85)	42.9(76)	22.1 (41)	31.9 (56)
Eating in front of TV	18.1(35)	14.4(26)	9.6 (18)	16.0 (28)
Eating out (3 or more times/week)	15.8(30)	17.7(32)	13.9 (26)	19.5 (34)
**Snacks consumption between meals**				
Chips	39.9(77)	41.4(75)	11.7 (22)	40.0 (70)
Chocolate	49.0(94)	51.9(94)	27.7 (52)	36.4 (64)
Soft drinks†	25.9(50)	39.8(72)	8.5 (16)	26.3 (46)
Sweetened drinks‡	64.2(124)	48.6(88)	43.6 (82)	52.8 (93)
Fruit	74.6(144)	62.4(113)	70.2 (132)	55.7 (98)
Sandwich	39.9(77)	40.3(73)	39.9 (75)	41.5 (73)
**Snacks bought from School shop**				
Chips	24.6(47)	29.8(54)	8.0 (15)	29.5 (52)
Chocolate	39.8(76)	48.1(87)	19.1 (36)	42.0 (74)
Soft drinks†	18.3(35)	24.3(44)	3.7 (7)	19.9 (35)
Sweetened drinks‡	50.8(97)	49.2(89)	35.6 (67)	52.8 (93)
Manoushe^	44.0(84)	52.5(95)	36.2 (68)	41.5 (73)
Croissant	34.6(66)	21.0(38)	18.6 (35)	21.6 (38)
**Physical Activity habit**				
Playing at recess	83.4 (161)	80.7 (146)	88.3 (166)	82.9 (145)
After school Physical activity/ week (at least once/week)	85.5 (164)	89.0 (161)	93.0 (176)	88.6 (154)
Playing at home afterschool	31.6 (61)	30.4 (55)	47.8 (89)	41.7 (73)
**Screen time habit**				
TV viewing during school days	31.2 (60)	29.3 (51)	30.1 (53)	32.2 (55)
TV viewing during week end	55.9 (108)	68.3 (123)	53.2 (100)	59.7 (104)
Electronic games during schooldays	59.0 (114)	59.6 (108)	61.1 (110)	56.0 (94)
Electronic games during weekend	48.4 (114)	50.0 (90)	49.2 (92)	49.5 (86)
**Knowledge and self-efficacy**				
***Determinant***	**Mean ± SD at Baseline**		**Mean ± SD Post Intervention**	
	**Intervention group (n = 193)**	**Control group (n = 181)**	**Intervention group (n = 193)**	**Control group (n = 181)**
**Knowledge score**	8.7 ± 3.0	8.9 ± 2.7	11.5 ± 3.0	8.5 ± 2.8
**Self-Efficacy score**	14.3 ± 2.7	13.8 ± 2.8	16.0 ± 2.6	13.7 ± 3.3

*Values derived from Chi Square & independent t-test. † Include carbonated beverages‡Include artificial juices and drinks^Lebanese pastry.*

The odds of an average student in an intervention school reporting a particular dietary habit at post-test compared to an average student in a control school was then calculated, controlling for the student’s baseline responses. As shown in Table 3, the odds of eating breakfast daily at post-test for an average student in the intervention group is 3.5 times greater than that of an average student in the control group, controlling for their baseline breakfast intake habits. The odds of eating in front of TV at post-test were 56% less likely in the intervention group compared with controls, again controlling for baseline habits (Table 3). The intervention was also successful at reducing the odds of having chips as snacks (OR = 0.14; CI = 0.11; 0.19) and the odds of drinking soft drinks (OR = 0.31; CI = 0.19; 0.52), but no additional differences were observed for other types of snacks such as fruits, chocolate, sweetened drinks and healthy sandwiches. Purchasing habits were also compared and results showed that the odds of buying chips (OR = 0.16; CI = 0.04; 0.61), soft drinks (OR = 0.12; CI = 0.04; 0.29) and chocolate (OR = 0.286; CI = 0.12; 0.66) were much less for an average student in the intervention versus control group, controlling for their baseline habits (Table 3).

**Table 3:** **Odds ratio comparing dietary habits, snack intake and snack purchase in an average student within an intervention school versus a control school at post-intervention, controlling for baseline measures†**

	Odds Ratio	95% CI
**Dietary habits** ***¥***		
Breakfast intake	3.50*	1.80; 6.90
Eating in front of TV	0.44*	0.23; 0.85
Number of snacks per day *£*	0.62	0.34; 1.15
Eating out per week *£*	0.70	0.35; 1.38
**Consumption of snacks between meals** ***¥***		
Chips	0.14*	0.11; 0.19
Chocolate	0.54	0.25; 1.15
Soft drinks‡	0.31*	0.18; 0.51
Sweetened drinks§	0.47	0.16; 1.40
Fruit	1.65	0.87; 3.10
Sandwich	1.50	0.78; 2.90
**Snacks bought from School shop** ***¥***		
Chips	0.16*	0.04, 0.61
Chocolate	0.29*	0.12; 0.66
Soft drinks‡	0.12*	0.04; 0.29
Sweetened drinks§	0.40	0.15; 1.07
Manoushe^	0.80	0.40; 1.50
Croissant	0.64	0.34; 1.12

†Baseline measure refers to the response provided at pre intervention *Significant at p < 0.05.

*‡Include carbonated beverages § Include artificial juices and drink. ^Lebanese pastry.*

*¥Reference group is “No” £ Reference group is “Less than 3”*.

### Changes in the physical activity and screen time habits

Students in the intervention group were 40% more likely to play at recess during post-test compared with control students, but changes in the levels of play at home, after school sports activity, and screen time habits, were not observed (Table 4).

**Table 4:** **Odds ratio comparing physical activity and sedentary habits in an average student within an intervention school versus a control school at post-intervention, controlling for baseline measures**†

Physical Activity Habit ***£***	Odds ratio	95% CI
Playing at recess	1.38*	1.10; 1.80
After school Physical activity per week	2.35	0.97; 5.65
Playing at home after school	0 .86	0.49; 1.52
**Screen time**		
TV viewing during school days ¥	0.86	0.50; 1.47
TV viewing during week end*§*	0.88	0.43; 1.80
Electronic games during schooldays*^*	1.32	0.75; 2.34
Electronic games during weekend*§*	1.05	0.35; 1.38

†Baseline measure refers to the response provided at pre-intervention. *Significant at p <0.05.

*¥Yes, No ( a lot coded as “yes”, a little and no coded as “No”), Reference “No”.*

*§A lot, a little (all day and twice a day coded as “a lot”, once a day and no, coded as “a little”), Reference “a little”.*

*^Everyday, not everyday (a little and a lot coded as “everyday”, 3 times a week and no, coded as “not everyday”), Reference “not everyday”.*

*£Reference group is “No”.*

### Changes in students’ knowledge and self-efficacy

The reported average knowledge score at baseline (range 0–14) was similar for the intervention group (8.7 ± 3.0) and control group (8.9 ± 2.7) at post-test; while the average score remained 8.5 ± 2.8 for control group students, it increased to 11.5 ± 3.0 for students in the intervention group. Controlling for baseline scores, the knowledge score increased on average by 2.86 units (95% CI = 1.7; 4.0; p < 0.001) for the students in the intervention group. With regards to self-efficacy scores (range 0–18), they were also similar at baseline for the intervention and control groups (14.3 ± 2.7 and 13.8 ± 2.8, respectively) and higher for the intervention group at post-test (16.0 ± 2.6 versus 13.7 ± 3.3 in control groups); thus, a 2.15 unit increase was observed (95% CI = 1.47; 2.82; p < 0.001) among students in the intervention group compared with those in the control group at post-test, controlling for baseline levels.

### Changes in anthropometric indices

The student’s body composition (height, weight, BMI, waist circumference) was similar in both groups at baseline (Table 1), and no significant changes in BMI (mean change for intervention group 0.37 ± 1.5, mean change for controls 0.19 ± 1.5) or waist circumference were observed in either group at post-test (p > 0.05).

### Student, parents and Teachers’ impressions of major programme facilitators and barriers

Table 5 presents some of the participants’ quotes from the focus groups. Overall, the facilitators that were recurrently mentioned and cited as being fundamental for the success of the intervention include: the attractive and fun activities during the educational lessons that motivated the students to increase their knowledge and skills in an enjoyable way; students’ active involvement in food preparation that improved their skills and self-confidence in choosing and preparing healthy foods; parental and teacher involvement in the intervention, emphasizing the importance of role modelling; and, the continued positive reinforcements via praise and tokens. Reported possible limitations to better outcomes and repeated themes included: the length of the intervention, which was considered by all too short duration to expect any major behavioural changes; continued availability of unhealthy food choices and lack of fruits and vegetables within the school environment; small playgrounds at schools and their unavailability near homes, as well as the high and competing attractiveness of computer and video games vis-à-vis physical activity.

**Table 5:** **Summary of quotes generated during the focus groups discussions**

Participants’ quotes	
	- “I am asking my mom to bake food instead of frying”
	- “Better if you come the whole year instead of just three months”
	- “I used to drink soft drinks with every meal, now I am having only half a cup a day”
	- “If the school shop offers fruits and fresh fruit juices, I would buy them instead of sweet drinks”
	- “The school shop should be closed or stop selling chips and sweetened drinks”
**Teachers**	- “They liked the sessions because it was not a lesson to memorize, though they memorized all messages”
	- “Hands-on activities helped convince the students with the information given in class”
	- “In spite of all your efforts, there was no cooperation from the school shop administrator”
**Parents**	- “They liked the idea that nothing is forbidden, as long as they eat the right servings of each food”
	- “My child wanted to go to school despite being sick with a high fever, she told me: today we have Mrs Carla (CHM) coming”
	- “Don’t know what you used or what was the method, but my boy was interested”
	- “Our kids need follow up; they forgot the healthy messages once the program was over”
	- “I cannot tell my boy not to buy from the shop when he sees his friends doing so”

## Discussion

‘Health-E-PALS’ is the first school-based intervention in Lebanese schools for the promotion of healthy eating and an active lifestyle. It is also the first study to demonstrate the feasibility of undertaking a successful school-based intervention despite the many contextual challenges that may be faced in a politically unstable context, with security threats and social unrests. The absence of a similar study from the region made it difficult for us to set benchmarks for what could be feasible and reasonable to accomplish. Notwithstanding these challenges, however, our pilot study illustrated that the intervention was culturally-relevant, promising and effective in improving many students’ dietary behaviours and determinants of behavioural change.

Health-E-PALS significantly increased students’ nutritional knowledge. Group discussions with students and parents revealed that this was mainly due to the classroom component being an activity rather than a didactic lesson. While increasing knowledge may be an important initial step, whether knowledge alone can directly translate to changes in behaviours remains questionable [16], which is why efforts to increase students’ self-efficacy is a fundamental part in personal change, as it motivates people to act [17]. Students exposed to the Health-E-PALS intervention scored higher on self-efficacy compared with controls. Self-efficacy is a stronger predictor of behavioural change, and has also been found to be an important mediator of the relation between knowledge and behaviour [18, 19].

With respect to behavioural outcomes, Health-E-PALS was effective in increasing the odds of daily breakfast intake, mostly that skipping meals, particularly breakfast, is a major risk factor for becoming overweight or obese as they grow older [20]. Daily intake of breakfast has been consistently linked to decreased BMI among children and adolescents, and a reduced risk of being overweight or obese [21]. Even if our finding were partially due to the lower proportion of children eating breakfast daily in the control group at post-test, qualitative findings confirm that breakfast intake was among the main healthy changes students incorporated into their dietary habits. Students in fact reported trying to “wake up early” in order to have breakfast on a regular basis and parents noted that their children were preparing breakfast following the examples given during the class sessions.

The lack of increase of fruits intake in our study corroborates others’ findings [22] and as such greater efforts must be made to promote the intake of fruits as a snack, dessert or part of a meal; worth noting however that the majority of students had already reported having fruits as a snack between meals. Reviews of studies that demonstrated a positive and statistically significant increase in fruit consumption noted that the effect size was small compared to the required intake [23]. However, availability and motivation were constructs that have been shown to mostly determine successful increases in fruit and vegetables consumption among children [24].

In the present pilot study, perceived self-efficacy, personal skills and food preferences were enhanced through food tasting workshops and snacks preparation, in line with evidence-based recommendations favouring tasting sessions of new healthy foods and drinks [25]. Interventions with parents further secured accessibility of these food items at home, and qualitative results showed that students were encouraged by their parents to consume fruits and vegetables, and that students were less resistant when offered these food items. Unfortunately, Health-E-PALS was not successful in changing the school environment, and the lack of fresh fruits and juices at the school shop was brought up during focus group interviews as one of the barriers to increased fruit intake, especially that students were willing “to buy fruits and fresh juices if offered at the school shop”. Lack of availability of healthy food options at school was also pointed among the perceived barriers to eating healthy in other studies looking at insights into children’s views on food and nutrition [26, 27].

Our programme also significantly decreased students’ intake and purchase of soft drinks but not of other sweetened drinks, as others have also found [28]. One possible explanation is the availability of sweetened beverages at school shops and at home as “healthy” substitutes for soft drinks. Parents and health educators mistakenly perceive them as a healthier option. Studies showed that reducing easy access to energy dense drinks could limit the chances of overconsumption [29].

Snacking has lately been suggested as being one of the causes of the increased energy intake responsible for the observed growing epidemic of obesity in the world [30]. Health-E-PALS effectively reduced students’ consumption and purchase of some types of energy dense snacks namely potato chips, which itself has been strongly linked to long-term weight gain [31]. One possible factor for this positive finding in our study might be the educational games’ emphasis on added fats and sugars in the common energy dense snacks. Though not statistically significant, the consumption and purchase of chocolate and biscuits also decreased. While access to potato chips was restricted in some schools, it was more difficult to convince the shop staff to remove all types of candies, as reported in other studies [32, 33]. Efforts to decrease availability of energy dense snacks and drinks were only successful in few schools; as shop owners were concerned about reduced profit. A recent review article on the effectiveness of school-based interventions in low- to middle-income countries found that changing the nutritional environment in schools poses a real challenge in these countries [34]. The Ministry of Higher Education in Lebanon has recently issued a law restricting the sale of competitive foods in school shops; this will hopefully improve the food environment in Lebanese elementary schools.

Health-E-PALS was not successful in raising the frequency of organised sports in schools curricula, though was effective in getting students engaged in after school sports at least once a week. Interviewed parents were reluctant to enrol their children in extracurricular activities due to budget, time, schedule constraints and homework overload, all of which have been previously reported in other studies [35]. In Lebanon, free physical activity facilities are scarce. After school sports are offered in some schools for an extra fee or in private sports clubs usually at an elevated monthly membership cost. Moreover, increasing expectations about academic achievements have prompted many Lebanese schools to cut back on both recess time and gym classes. Health-E-PALS also emphasized the importance of unorganised physical activity through several means one of which is the pedometer workshop that proved to be successful in motivating children to move. Pedometer-based interventions are becoming more popular as a low-cost and effective method for promoting physical activity and increasing walking [36–38]. Group discussions unanimously pointed towards pedometers being an incentive to increase daily physical activity, as others have found [39]. Health-E-PALS also positively influenced play during recess but not at home, probably due to unsafe neighbourhoods as explained by parents during the focus group discussions. The built environment in the city of Beirut prevents children from engaging in spontaneous physical activity and play. Improving the urban environments is not one of the Lebanese government’s priorities, which mainly revolves around economic and political problems. Thus, short-term solutions should focus on improving the school environments by regarding the PE session as a central part of the student’s wellbeing.

Mounting evidence shows that independent of physical activity levels; sedentary behaviours are associated with increased risk for many diseases and namely adiposity [40–42]. Intervention studies which helped children decrease their sedentary time reported desirable changes in body weight and BMI [43]. Health-E-PALS did not achieve a decrease in students’ screen time habits, despite the strict rules at home for screen time during school days in some families, as reported during focus groups. Generally, parents perceived reducing their children’s TV viewing as a difficult task [44] and longer follow-ups are needed to observe changes [45].

The lack of any observed changes in students’ BMI and waist circumference measurements in the present study is probably due to the insufficient duration between pre- and post-test (i.e.12 weeks), making it difficult to detect any noticeable changes in adiposity. Systematic reviews [10] showed that the majority of studies targeting six to 12 year old children that proved effective on some indicators of adiposity, involved long-term intervention periods (>12 months). Nonetheless, our study positively impacted major mediating variables, as well as some behavioural habits.

Since parents are fundamental to the success of any child intervention, parents were involved in Health-E-PALS both through direct (via meetings and health fairs) and indirect exposure to the programme’s elements, by sending information packets and recipes home with the students. It is worth noting that direct methods to engage parents have proven to be more successful than indirect methods [46] although the best way to involve parents is yet unknown. Parents helped in the success of the Health-E-PALS intervention by ensuring the availability and accessibility of healthier food options at home, and through role modelling. One of the challenges faced with parents however is their attendance in meetings, which was poor in some schools. Inviting parents to attend their children’s’ performances, and providing meals and tokens helped secure parents’ participation in the present study, as suggested elsewhere [47].

Our pilot evaluation of the Health-E-PALS, including culturally sensitive and innovative components, proved successful in increasing students’ nutritional knowledge and self-efficacy, as well as improving various important behavioural risk factors to obesity. The traditional foods, rhymes, riddles and games helped children relate messages to their daily routine and local environment. Contextual influences such as social values and cultural norms were important parameters to include for the success of school-based interventions in low and middle income countries [34]. Holding parents’ meetings around breakfast and demonstrating how traditional foods can be served in a healthy way was key to this context since gathering around meals is intrinsic to the Lebanese culture. This secured better attendance during meetings especially with families of low socioeconomic status. Health-E-PALS was generally well accepted by students, teachers and their parents. The focus group discussions were fundamental for soliciting important feedback and verifying the intervention was indeed tailored to the Lebanese culture and context. This essential component was a driving force for the positive findings achieved in the present study. Furthermore, given this particular social and cultural context the applicability and transferability of the present study to other countries of the region ought to be considered.

Despite the positive findings and study’s contribution, the results must be interpreted in light of some limitations including: the purposeful sampling of schools who were keen to take part in the study, despite the fact they were matched and randomised to intervention and control group, may have increased compliance with the intervention; the relatively low number of schools (eight) that were randomised may have limited the likelihood of distributing potential confounders equally between the intervention and control schools; the relatively short study duration (three months), which may not have been sufficient to induce proper and sustained behaviour change; failure to succeed in modifying the school’s food environment due to lobbying and lack of support of some of the school authorities. Several challenges were also faced during implementation of the programme given Lebanon’s political and social unrest; extra sessions were necessary to make up for the missed ones that were canceled due to unpredicted strikes and security events prevailing in the country. The health fair event that was cancelled in one of the schools could not be rescheduled as end of year final exams had begun. Nonetheless, despite these field constraints, the Health-E-PALS programme was successfully delivered as designed: the 12 educational lessons and activities were all implemented in the intervention schools along with meetings with parents and the food service management.

## Conclusion

Health-E-PALS showed that this culturally appropriate, theory-based intervention that used a whole school approach and included interactive learning was feasible and increased students’ nutritional knowledge and self-efficacy, and decreased their purchase and consumption of high energy dense snacks and beverages. Next steps entail planning for a larger scale evaluation of this intervention, to include a greater number of schools from different regions of Lebanon, for a longer duration period. The large scale trial should emphasise the physical education component, improve the quality of foods available to students in school shops, and be delivered by trained school personnel, for better sustainability of the programme.

## Electronic supplementary material

Additional file 1: **PDF document, Showing the Student questionnaire used in this study, uploaded separately.** (PDF 805 KB)

Additional file 2: **PDF document presenting a table with recoded variables used for the analysis of this study, uploaded separately.** (PDF 147 KB)

## References

[1] Musaiger AO (2011). Overweight and obesity in eastern Mediterranean region: prevalence and possible causes. J Obes.

[2] Food and Agriculture Organization (2007). Lebanese Republic Nutrition Profile. Nutrition and Consumer Protection Division.

[3] Nasreddine L, Naja F, Akl C, Adra N, Sibai A, Hwalla N (2012). Prevalence and Determinants of Overweight and obesity in a National Sample of 5–12 Years Old Lebanese Children.

[4] Akl C (2012). Prevalence and Determinants of Overweight and Obesity in a Nationally Representative Sample of Lebanese Children 5 to 12 years old.

[5] World Health Organization (2011). Lebanon, 2011, Global School-based Student Health Survey (GSHS).

[6] Chakar HR, Salameh P (2011). Public schools adolescents’ obesity and growth curves in Lebanon. LMJ.

[7] Fazah A, Jacob C, Moussa E, El-Hage R, Youssef H, Delamarche P (2010). Activity, inactivity and quality of life among Lebanese adolescents. Pediatr Int.

[8] Sibai A, Hwalla N, Adra N, Rahal B (2003). Prevalence and covariates of obesity in Lebanon: findings from the first epidemiological study. Obes Res.

[9] Nasreddine L, Naja F, Akl C, Adra N, Sibai A, Hwalla N (2012). Nutrient intakes among 5–12 years old Lebanese children: A cross-sectional national survey.

[10] Waters E, De Silva-Sanigorski A, Hall B, Brown T, Campbell KJ, Gao Y, Armstrong R, Prosser L, Summerbell CD (2011). Interventions for preventing obesity in children. Cochrane Database Syst Rev.

[11] Shaya FT, Flores D, Gbarayor CM, Wang J (2008). School-based obesity interventions: a literature review. J Sch Health.

[12] Summerbell CD, Waters E, Edmunds LD, Kelly S, Brown T, Campbell KJ (2008). Interventions for preventing obesity in children (Review). Cochrane Database Syst Rev.

[13] Habib-Mourad C, Moore H, Nabhani-Zeidan M, Hwalla N, Summerbell C (2014). Health-E-PALS: promoting Healthy Eating and Physical Activity in Lebanese school children – Intervention development. Educ Health.

[14] Bandura A (1986). Social Foundations of Thought and Action: A Social Cognitive Theory.

[15] Burnard P (1991). A method of analyzing interview transcripts in qualitative research. Nurse Educ Today.

[16] Harrison M, Burns CF, Mc Guinness M, Heslin J, Murhpy NM (2006). Influence of a health education intervention on physical activity and screen time in primary school children: “Switch Off-Get Active.”. J Sci Med Sport.

[17] Swaminathan S, Thomas T, Kupard AV, Vaz M (2009). Perception of healthy eating: A qualitative study of school-going children in south India. Health Educ J.

[18] Bandura A (2004). Health promotion by social cognitive means. Health Educ Behav.

[19] Rimal RN (2000). Closing the knowledge-behavior gap in health promotion: the mediating role of self-efficacy. Health Commun.

[20] Nasreddine L, Naja F, Chamieh MC, Adra N, Sibai A, Hwalla N (2012). Trends in overweight and obesity in Lebanon: evidence from two national cross-sectional surveys (1997 and 2009). BMC Public Health.

[21] Szajewska H, Ruszczyński M (2010). Systematic review demonstrating that breakfast consumption influences body weight outcomes in children and adolescents in Europe. Crit Rev Food Sci Nutr.

[22] Van Horn L, Obarzanek E, Friedman L, Gernhofer N, Barton B (2005). Children’s Adaptations to a Fat-Reduced Diet. Dietary Intervent Stud Child (DISC).

[23] Thomson CA, Ravia J (2011). A systematic review of behavioral interventions to promote intake of fruit and vegetables. J Am Diet Assoc.

[24] Reynolds KD, Hinton AW, Shewchuk RM, Hickey CA (1999). Social cognitive model of fruit and vegetable consumption in elementary school children. J Nutr Educ.

[25] Summerbell CD, Moore HJ, Vögele C, Kreichauf S, Wildgruber A, Manios Y, Douthwaite W, Nixon CA, Gibson EL, ToyBox-study group (2012). Evidence-based recommendations for the development of obesity prevention programs targeted at preschool children. Obes Rev.

[26] Shepherd J, Harden A, Rees R, Brunton G, Gracia J, Oliver S, Oakley A (2006). Young people and healthy eating: a systematic review of research on barriers and facilitators. Theory Pract.

[27] McKinley MC, Lowis C, Robson PJ, Wallace JMW, Morrissey M, Moran A, Livingstone MBE (2005). It’s good to talk: children’s views on food and nutrition. Eur J Clin Nutr.

[28] Sichieri R, Paula Trotte A, De Souza RA, Veiga GV (2009). School randomized trial on prevention of excessive weight gain by discouraging students from drinking sodas. Public Health Nutr.

[29] Fernandes MM (2008). The effect of soft drink availability in elementary schools on consumption. J Am Diet Assoc.

[30] Piernas C, Popkin BM (2010). Trends in snacking among U.S. children. Health Affairs.

[31] Mozaffarian D, Hao T, Rimm EB, Willett WC, Hu FB (2011). Changes in diet and lifestyle and long-term weight gain in women and men. New Engl J Med.

[32] Marcus C, Nyberg G, Nordenfelt A, Karpmyr M, Kowalski J, Ekelund U (2009). A 4-year, cluster-randomized, controlled childhood obesity prevention study: STOPP. Int J Obes.

[33] Fernandes PS, Bernardo Cde O, Campos RM, Vasconcelos FA (2009). Evaluating the effect of nutritional education on the prevalence of overweight/obesity and on foods eaten at primary schools. J Pediatr (Rio J).

[34] Verstraeten R, Roberfroid D, Lachat C, Lefroy JL, Holdsworth M, Maes L, Kolsteren PW (2012). Effectiveness of preventive school-based obesity interventions in low-and middle-income countries: a systematic review. Am J Clin Nutr.

[35] Hesketh K, Waters E, Green J, Salmon L, Williams J (2005). Healthy eating, activity and obesity prevention: a qualitative study of parent and child perception in Australia. Health Promot Int.

[36] Chan CB, Ryan DAJ, Tudor-Locke (2004). Health benefits of a pedometer-based physical activity intervention in sedentary workers. Prev Med.

[37] Croteau KA (2004). A preliminary study on the impact of a pedometer-based intervention on daily steps. Am J Health Promot.

[38] Schofield L, Mummery WK, Schofield G (2005). Effects of a controlled pedometer-intervention trial for low-active adolescent girls. Med Sci Sports Exerc.

[39] Heesch KC, Dinger MK, Mcclary KR, Rice KR (2005). Experiences of Women in a minimal contact pedometer-based intervention: A qualitative study. J Womens Health.

[40] Treuth MS, Catellier DJ, Schimtz KH, Pate RR, Elder JP, Murray RG, Blew RM, Yang S, Webber L (2007). Weekend and weekday patterns of physical activity in overweight and normal-weight adolescent girls. Obesity.

[41] Katzmarzyk PT, Church TS, Craig CL, Bouchard C (2009). **Sitting time and mortality from all causes, cardiovascular disease, and cance**r. Med Sci Sports Exerc.

[42] Owen N, Bauman A, Brown W (2009). Too much sitting: a novel and important predictor of chronic disease risk?. Br J Sports Med.

[43] Tremblay MS, LeBlanc AG, Kho ME, Saunders TJ, Larouche R, Colley RC, Goldfield G, Gorber SC (2011). Systematic review of sedentary behaviour and health indicators in school-aged children and youth. Int J Behav Nutr Phys Act.

[44] Dorey E, Roberts V, Maddison R, Meagher-Lundverg P, Dixon R, Ni Mhurchu C (2009). Children and television watching: a qualitative study of New Zealand parents’ perceptions and views. Child Care Health Dev.

[45] Cerin E, Barnett A, Baranowski T (2009). Testing theories of dietary behavior change in youth using the mediating variable model with intervention programs. J Nutr Educ Behav.

[46] Hingle MD, O’Connor TM, Dave JM, Baranowski T (2010). Parental involvement in interventions to improve child dietary intake: A systematic review. Prev Med.

[47] Pocock M, Trivedi D, Wills W, Bunn F, Magnusson J (2010). Parental perceptions regarding healthy behaviours for preventing overweight and obesity in young children: a systematic review of qualitative studies. Obesity Reviews.

[CR48] The pre-publication history for this paper can be accessed here:http://www.biomedcentral.com/1471-2458/14/940/prepub

