# Supplementary material for: Promoting healthy eating and physical activity among school children: findings from Health-E-PALS, the first pilot intervention from Lebanon
Source: BMC Public Health. 2014 Sep 10;14:940. doi: 10.1186/1471-2458-14-940 (PMC4167260; doi:10.1186/1471-2458-14-940)

File 1: Final Questionnaire

STUDENT QUESTIONNAIRE

YOUR HEALTHY HABITS

School Name:

Student name:

Date:

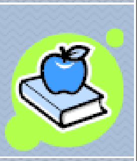

Dear student,

Thank you for taking the time to fill the attached questionnaire.

- The following questions are about your eating and exercise habits
- Please fill in your answers as honestly as possible
- If you find difficulties with any of the questions, do not hesitate to ask for help.

Best of luck,

Carla

Carla Habib Mourad

Circle the answer that mostly fits with your daily habits.

1- **Do you usually have breakfast?**
   1) Never
   2) Sometimes
   3) Yes, every day

2- **Do you usually have lunch?**
   1) Never
   2) Sometimes
   3) Yes, every day

3- **Do you usually have dinner?**
   1) Never
   2) Sometimes
   3) Yes, every day

4- **Do you usually have snacks between your meals?**
   1) Yes
   2) No

5- **if yes, do you take any of these snacks between your meals (at school or at home)?**
   1) fruits
   2) chocolate or cookies
   3) soft drinks
   4) juice
   5) chips
   6) cheese sandwich

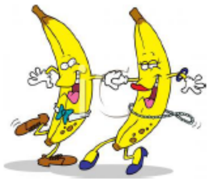

6- **How many snacks do you have a day?**

1) One
2) Two
3) Three or more

7- **Do you buy food from your school shop?**

1) yes
2) no

8- **What do you buy from your school shop?**

1) Chocolate or cookies
2) Juice
3) Soft drinks
4) Water
5) Croissant
6) Manoushe
7) Chips or peanuts

9- **Do you bring food with you to school from home?**

1) Yes
2) No

10- **Do you choose what to put in your lunch box?**

1) Yes
2) No
3) Sometimes

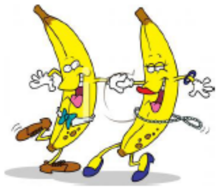

11- How many times a week you eat outside home or have a delivery meal?

1) 3 or more times per week
2) Twice per week
3) Once per week
4) Never

12- Do you choose some of the foods your parents buy from the supermarket?

1) Yes
2) No

13- Do you watch TV during school days?

1) I watch TV a lot everyday
2) I watch TV a little before I go to sleep
3) I don't watch TV

14- Do you watch TV during week- ends?

1) All day
2) Twice a day
3) Once a day
4) I don't watch TV

15- Do you eat while watching TV?

1) All the time
2) Sometimes
3) No

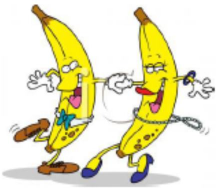

16- What do you play at school during recess?

1) I don’t play
2) I play ball games
3) Jumping rope
4) I run or play “catch me if you can”
5) Other

17- Do you play at home after school?

1) Yes
2) No
3) Sometimes

18- If yes, what do you play at home after school?

1) I don’t play
2) I play ball games
3) I ride my bike or my rollers/skates

19- How many days a week do you have sports/exercise sessions at school?

1) Not one day
2) One day
3) Two days
4) More than three

20- Do you participate in the exercise sessions at school?

1) I don’t participate at all
2) Sometimes
3) Yes, every time

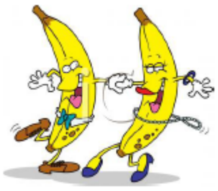

21- How many times per week do you do sports (Football, Basket Ball, Dance, Judo, Swimming…) after school or during the week-end?

1) I don't
2) Once per week
3) 2 times per week
4) 3 times or more

22- Do you play computer games; Play station, PSP, Nintendo after school?

1) Every day for a long time
2) Every day for a short time
3) 2-3 times a week
4) I don't play

23- Do you play computer games; Play station, PSP, Nintendo during week-ends? (You can choose more than one answer)

1) All day
2) Twice a day
3) Once a day
4) I don't play

24- From which type of food you should eat the *least*?(choose only one type of food)

1) Bread, rice and pasta
2) Milk, cheese and yogurt
3) Sweets, fats and oils
4) Fruits and vegetables
5) Meat, chicken and eggs
6) I don't know

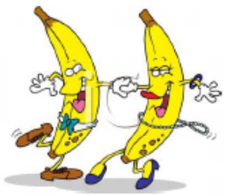

25- **From which type of food you should eat the *most*?(choose <u>only one</u>)**

1) Bread, rice and pasta
2) Milk, cheese and yogurt
3) Sweets, fats and oils
4) Fruits and vegetables
5) Meat, chicken and eggs
6) I don't know

26- **How many servings of fruits and vegetables you should have a day?**

1) One
2) 2 or 3
3) 4 or 5
4) 5 or more
5) I don't know

27- **Eating breakfast helps me do well in class.**

1) Yes always
2) Sometimes
3) No, never

28- **The best fluid for my body is: *(choose one answer)***

1) Water
2) Juice
3) Soft drinks
4) I don't know

29- **Juice and soft drinks cause dental caries.**

1) Yes
2) No
3) I don't know

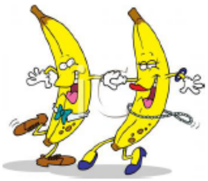

30- Chocolate and candies cause dental caries.

1) Yes
2) No
3) I don't know

31- Which of these foods have less fat?

1) fried potatoes
2) baked potatoes
3) I don't know

32 - Which of these foods have less fat?

1) croissant
2) corn flakes
3) I don't know

33- Which of these foods have less fat?

1) chips
2) pop corn
3) I don't know

34-Which of these foods have less sugar?

1) soft drinks
2) Milk
3) I don't know

35 - Which of these foods have less sugar?

1) doughnuts
2) corn flakes
3) I don't know

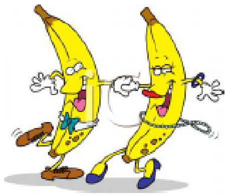

36 - Which of these foods have less sugar?

1) fresh juice
2) tang (artificial juice)
3) I don't know

37-For my health, I should exercise :

1) every day
2) once a week
3) twice a week
4) I don't know

38- How sure are you that you can prepare a healthy breakfast by yourself?

1) Very sure
2) Little sure
3) Not sure

39 - How sure are you that you can prepare a healthy snack by yourself?

1) Very sure
2) Little sure
3) Not sure

40 -How sure are you that you can drink less sweet and soft drinks?

1) Very sure
2) Little sure
3) Not sure

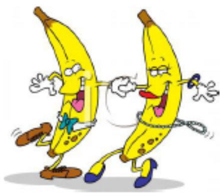

41 -How sure are you that you can do more sports during the week?

1) Very sure
2) Little sure
3) Not sure

42 -How sure are you that you can eat more fruits a day?

1) Very sure
2) Little sure
3) Not sure

43 -How sure are you that you can eat more vegetables a day?

1) Very sure
2) Little sure
3) Not sure

44 -How sure are you that you can eat breakfast every day?

1) Very sure
2) Little sure
3) Not sure

45 -How sure are you that you can eat a fruit instead of another snack during the day?

1) Very sure
2) Little sure
3) Not sure

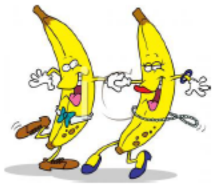

46-How sure are you that you can spend less time playing computer and PlayStation games?

1) Very sure
2) Little sure
3) Not sure

47 -The food I eat can affect my health.

1) Yes
2) No
3) I don't know

48 -The foods that I eat now are healthy.

1) Yes
2) No
3) I don't know

49 -People who weigh more than they should, may have health problems

1) Yes
2) No
3) I don't know

Thank you 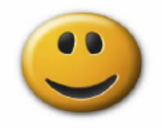

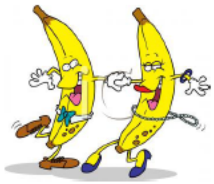

Supplement: Supplementary file 1 — Additional file 1: PDF document, Showing the Student questionnaire used in this study, uploaded separately. (PDF 805 KB) [file 12889_2014_7033_MOESM1_ESM.pdf]
