# Supplementary material for: Promoting healthy eating and physical activity among school children: findings from Health-E-PALS, the first pilot intervention from Lebanon
Source: BMC Public Health. 2014 Sep 10;14:940. doi: 10.1186/1471-2458-14-940 (PMC4167260; doi:10.1186/1471-2458-14-940)
Supplement: Supplementary file 2 — Additional file 2: PDF document presenting a table with recoded variables used for the analysis of this study, uploaded separately. (PDF 147 KB) [file 12889_2014_7033_MOESM2_ESM.pdf]

File 2: Variables recoded

Variable name	Question	Variable recoding
Breakfast intake	Do you usually have breakfast: everyday, sometimes, I don't	Yes, No (sometimes and I don't coded as 'No")
Number of snacks per day	How many snacks do you have a day: once, twice, 3 or more	Once or twice, 3 or more
Eating out	How many times a week do you eat out: once or twice, 3 or more, never	Sometimes, 3 or more (never and once or twice coded as "sometimes")
Eating in front of TV	Do you eat in front of TV: yes, sometimes, No	Yes, No (sometimes and No coded as "No")
TV viewing during week days	Do you watch TV during weekdays: a little, a lot, No	Yes, No (a lot coded as "yes", a little and no coded as "No")
TV viewing during week ends	Do you watch TV during weekends: all day, once, twice, No	A lot, a little (all day and twice a day coded as "a lot", once a day and no, coded as "a little")
Electronic games during week days	Do you play computer games during weekdays: a little, a lot,3 times a week, No	Everyday, not everyday (a little and a lot coded as "everyday", 3 times a week and no, coded as "not everyday")
Electronic games during week ends	Do you play computer games during weekends: once a day, twice a day, all day, No	A lot, a little (all day and twice a day coded as "a lot", once a day and no coded as "a little")
Physical activity during recess	What do you play during recess: ball games, jumping rope, running games, You don't	Play, don't play (ball games, jumping rope and running games coded as "play")
Physical activity at home	What do you play at home: ball games, rollers, bicycle, you don't	Play, don't play (ball games, rollers and bicycle coded as "play")
After school physical activity	How many times a week you do sports after school: once, twice, 3 or more, No	No, At least once (once, twice and 3 or more coded as "at least once per week")
Health belief (1)	The food you eat can affect your health: yes, no, don't know	Yes, No (don't know and No coded as 'No")
Health belief (2)	The foods you eat now are healthy: yes, no, don't know	Yes, No (don't know and No coded as 'No")
Health belief (3)	People who weigh more may have health problems: yes, no, don't know	Yes ,No (don't know and No coded as 'No")
